# Chaperonin‐containing TCP‐1 subunit 2‐mediated aggrephagy: A potential target for treating neurodegeneration

**DOI:** 10.1002/ctm2.1027

**Published:** 2022-08-21

**Authors:** Xueshen Chen, Min Zhang

**Affiliations:** ^1^ School of Pharmaceutical Sciences Tsinghua University Beijing China

1

One of the major pathological features of neurodegenerative diseases is protein aggregation, which is frequently accompanied by neuronal loss and neuronal degeneration in many disease‐specific brain areas. For example, the two main neuropathological lesions in Alzheimer's disease are plaques containing β‐amyloid peptide and neurogenic fibre tangles consisting of hyperphosphorylated tau. In patients with Parkinson's disease, Lewy bodies made of α‐synuclein are frequently observed in the brain. Similarly, in patients with Huntington's disease, which is caused by a mutation in a single gene, Huntingtin, expansion of a glutamine‐encoding repeat in the first exon leads to Huntingtin (HTT) protein aggregation. In addition, aggregation of multiple proteins (e.g., SOD1, FUS, and TDP‐43) has been reported in the pathogenesis of amyotrophic lateral sclerosis.^3^

Researchers have speculated that autophagy, an essential process for the removal of protein aggregates, may underlie neurodegenerative diseases. Autophagy is a bulk degradative process in which intracellular contents, such as misfolded proteins and damaged organelles, are degraded by the lysosome and recycled to maintain normal cellular homeostasis. A class of autophagic receptor proteins selectively recognizes and facilitates the autophagic destruction of protein aggregates through a selective autophagy pathway, also known as “aggrephagy”.^5^ Accumulating evidence indicates that deficiency of selective autophagy is involved in a range of neurodegenerative disorders and that restoring its function can stop the spread of disease.^8^

Ma et al. recently discovered an autophagy receptor that can facilitate the targeting of the autophagic membrane to protein aggregates and the removal of proteins that are prone to aggregation.^6^ Using an in vitro reconstitution system to recapitulate autophagy recognition of protein aggregates, they identified multiple chaperone proteins that promoted the clearance of the aggregation‐prone polyglutamine (polyQ)‐HTT protein. Amongst the chaperones, the TRiC subunit chaperonin‐containing TCP‐1 subunit 2 (CCT2) had the most potent effect and was further studied. They found that CCT2 expression increased the clearance of multiple aggregation‐prone proteins involved in neurodegeneration, including Tau (P301L), SOD1 (G93A), FUS (P525L), and polyQ‐HTT. They also confirmed the effect of CCT2‐enhanced clearance of polyQ‐HTT in brains in a mouse model of Huntington's disease (polyQ [Q140] knockin).

Protein aggregation occurs through a phase transition process in which the aggregation‐prone proteins form a liquid granule via phase separation, which has been considered physiological. Thereafter, the granule gradually ages to form a solid aggregate, or pathological deposit, in the cell. Previous studies have found that selective autophagy preferentially removes protein aggregates that exhibit some liquidity.^7^, ^10^ Interestingly, the authors show that the ubiquitin‐binding receptors P62, NBR1 and TAX1BP1 selectively clear liquid FUS (P525L). However, CCT2 prefers to remove cargos with little liquidity, and this preference is not due to alteration of the liquid‐to‐solid transition of the cargos. Given that solid aggregates are potentially dangerous components, the recognition and clearance of solid protein aggregates by CCT2‐mediated aggrephagy may give CCT2 a unique significance in rescuing the disease process in neurodegenerative disorders.

In addition to functioning in aggrephagy, CCT2 is also a component of the chaperone complex TRiC, which maintains proteostasis by preventing protein folding and aggregation.^4^ For example, it has been demonstrated that TRiC prevents the aggregation of mutant HTT proteins.^9^ Therefore, expression of CCT2 may provide a dual beneficial effect—aggregation prevention and clearance (Figure 1). In fact, a recent study found a decrease in CCT2 levels during the aging process and in multiple neurodegenerative diseases.^1^ This evidence supports the notion that restoration of CCT2 may be a potential strategy for preventing neurodegeneration associated with protein aggregation. Current treatments for neurodegenerative diseases include traditional pharmacological treatments, gene therapy, antibody‐induced immunotherapy, and nanotechnology, few of which can reverse the clinical symptoms in higher severity patients.^2^ CCT2's full involvement in preventing and clearing toxic protein aggregates makes it an enticing therapeutic target against neurodegeneration and other related diseases (Figure 2).

**FIGURE 1:**
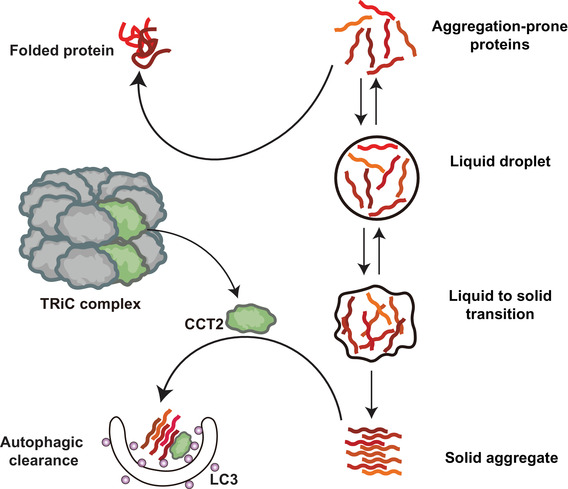
Chaperonin‐containing TCP‐1 subunit 2 (CCT2)’s dual function in preventing protein aggregation and removing protein aggregates. CCT2 can function as a component of the TRiC complex to suppress protein misfolding and aggregation; also, it can act as a monomeric autophagy receptor to facilitate the clearance of solid protein aggregates by selective autophagy

**FIGURE 2:**
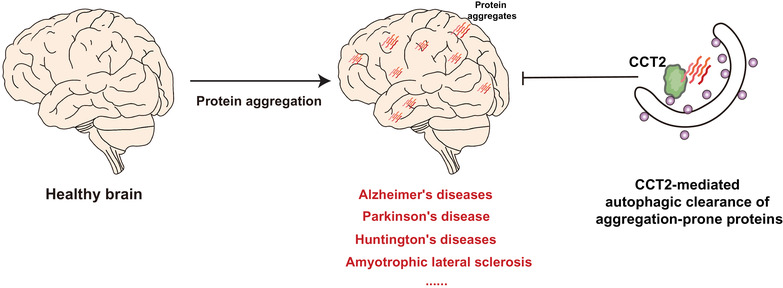
Chaperonin‐containing TCP‐1 subunit 2 (CCT2) as a potential target against neurodegenerative diseases caused by protein aggregation. Protein aggregation is a pathological feature of several neurodegenerative diseases. The function of CCT2 in clearing aggregation‐prone proteins through selective autophagy makes it a potential target for the treatment of neurodegenerative diseases

The findings by Ma et al. provide a potential new focus for the treatment of neurodegeneration. Clinical trials and drug development for neurodegenerative disorders have often failed because of the complex pathophysiological changes occurring during the disease process; therefore, more in vivo experimental evidence is needed to determine whether CCT2 can be a potential therapeutic target against neurodegenerative diseases.

## References

[1] Brehme M , Voisine C , Rolland T , et al. A chaperome subnetwork safeguards proteostasis in aging and neurodegenerative disease. Cell Rep. 2014;9:1135‐1150. 25437566 10.1016/j.celrep.2014.09.042 PMC4255334

[2] Budd Haeberlein SL , Harris TJ . Promising targets for the treatment of neurodegenerative diseases. Clin Pharmacol Ther. 2015;98:492‐501. 26250447 10.1002/cpt.195

[3] Davis AA , Leyns CEG , Holtzman, DM . Intercellular spread of protein aggregates in neurodegenerative disease. Annu Rev Cell Dev Biol. 2018;34:545‐568. 30044648 10.1146/annurev-cellbio-100617-062636 PMC6350082

[4] Jin M , Liu C , Han W , Cong Y . TRiC/CCT chaperonin: Structure and function. Subcell Biochem. 2019;93:625‐654. 31939165 10.1007/978-3-030-28151-9_19

[5] Lamark T , Johansen T . Aggrephagy: Selective disposal of protein aggregates by macroautophagy. Int J Cell Biol. 2012;2012:736905. 22518139 10.1155/2012/736905 PMC3320095

[6] Ma X , Lu C , Chen Y , et al. CCT2 is an aggrephagy receptor for clearance of solid protein aggregates. Cell. 2022;185:1325‐1345. 35366418 10.1016/j.cell.2022.03.005

[7] Noda NN , Wang Z , Zhang H . Liquid−liquid phase separation in autophagy. J Cell Biol. 2020;219:e202004062. 32603410 10.1083/jcb.202004062 PMC7401820

[8] Scrivo A , Bourdenx M , Pampliega O , Cuervo AM . Selective autophagy as a potential therapeutic target for neurodegenerative disorders. Lancet Neurol. 2018;17:802‐815. 30129476 10.1016/S1474-4422(18)30238-2 PMC6359907

[9] Shahmoradian SH , Galaz‐Montoya JG , Schmid, MF , et al . TRiC's tricks inhibit huntingtin aggregation. Elife. 2013;2:e00710. 23853712 10.7554/eLife.00710 PMC3707056

[10] Sun D , Wu R , Li P , Yu L . Phase separation in regulation of aggrephagy. J Mol Biol. 2020;432:160‐169. 31260696 10.1016/j.jmb.2019.06.026

